# Effect of Electrode Positioning on Electrokinetic Remediation of Contaminated Soft Clay with Surface Electrolyte

**DOI:** 10.3390/toxics12100758

**Published:** 2024-10-19

**Authors:** Zhaohua Sun, Shuwen Xu, Jianming Zhang, Beukes Demarscho Eugene, Sheng Li

**Affiliations:** 1 School of Transportation and Civil Engineering, Nantong University, Nantong 226019, China; 15962818282@163.com (S.X.); beukesde@gmail.com (B.D.E.); lisheng202409@163.com (S.L.); 2 Key Laboratory of New Technology for Construction of Cities in Mountain Area, Ministry of Education, Chongqing University, Chongqing 400045, China; 3 Zhongru Construction Group Co., Ltd., Nantong 226199, China; 18862755013@163.com

**Keywords:** electrokinetic remediation, soft clay, electrode position, electrolyte, removal efficiency

## Abstract

Soft clay contamination is an increasingly global issue with significant implications for land development and human health. Electrokinetic remediation (EKR) has demonstrated significant potential for cleaning contaminated soils. It is crucial to develop efficient processes that minimize environmental impact and reduce costs. A series of citric acid (CA)-enhanced EKR tests were conducted using a novel experimental setup, with the electrolyte positioned above the soil surface, to examine the impact of four different electrode arrangements on the effectiveness of EKR. The position of the electrode end had a significant impact on the migration of ions in the anolyte and catholyte, which in turn affected the volume reduction in the anolyte, the magnitude of the current, and the migration of heavy metals. The electrode arrangement mode *c* (electrodes suspended in the electrolytes) can enhance the migration of the anolyte and reduce the drainage of the soil, making it an effective measure for improving the removal rate of heavy metals. After the heavy metal remediation is complete, the bearing capacity of the soil should be increased. Changing the electrode arrangement to mode *d* (anode suspended in the anolyte, a very small part of the cathode inserted into the soil) is an effective measure for reducing the soil water content and improving soil strength.

## 1. Introduction

Electrokinetic remediation (EKR) of contaminated soil is not only a vital tool for environmental protection but also a crucial technology for improving land use efficiency and ensuring public health [1]. This method leverages electric currents to mobilize and remove pollutants from the soil, and it offers high adaptability to various contaminants, including heavy metals and organic compounds [2,3]. As an advanced environmental protection technology, electrokinetic remediation effectively extracts harmful substances from the soil, thereby reducing the risk of groundwater and air pollution and safeguarding ecosystems. Moreover, it plays a pivotal role in restoring soil health, enhancing soil quality, and making the land viable again for agriculture and other purposes. This approach promotes sustainable land use while preventing contaminants from entering the food chain or posing other threats to human health [4].

The EKR efficiency in contaminated soil is affected by a range of factors, such as the type and concentration of contaminants; soil characteristics; electrode configurations; electric field strength and current density; soil pH and chemical composition; and the duration and specific operational conditions of the remediation process [5,6]. Extensive research has been conducted on these influencing factors, leading to the development of numerous valuable insights. In particular, studies on electrode configurations have concentrated on various aspects, including electrode arrangements for generating different electroosmotic flow directions, polarity reversals, electrode spacing, injection wells, and the elimination of metal debris [7]. Moon et al. [8] tested different electrode configurations during the EK-PRB process for trichloroethylene-contaminated sites under hydro-geo-chemical conditions. However, the precise positioning of electrodes within the electrolyte—such as whether they make direct contact with the soil or the volume of electrolyte between the electrodes and the soil—remains underexplored in terms of their impact on the EKR efficiency. Furthermore, for contaminated soils with high water content and low bearing capacity, it is crucial to focus on reducing drainage or enhancing bearing capacity, which could facilitate further development and practical application of these remediation techniques.

In laboratory experiments, the EKR setup typically consists of three compartments: a central soil compartment flanked by an anode compartment on one side and a cathode compartment on the other. The soil compartment is filled with layered and compacted soil, either artificially or naturally contaminated. The anode and cathode compartments are subsequently filled with electrolyte solutions. The positions of the electrodes in the experimental setup vary. In some studies, the electrodes were in direct contact with the soil [9,10], while in others, a filter paper and a porous stone were placed between the electrodes and the soil [11,12]. Additionally, in some studies, the electrodes were placed in an electrolyte solution without direct contact with the soil [13,14,15]. The electrode shapes used in studies have included planar electrodes [14], rod-shaped electrodes [16], and mesh electrodes [13,17]. It is worth investigating whether the relative positions of different electrode shapes in relation to the contaminated soil affect the efficiency of electrokinetic remediation.

The influence of the scale-up, both in technical and engineering aspects, as well as in the behavior of different variables during the EKR process, was studied. In a pilot-scale project conducted in South Korea, porous column cathodes and anodes were installed on the top and bottom surfaces of 0.6-m-thick contaminated soil. Water and citric acid (CA) solutions were continuously supplied through the perforated electrodes to keep the soil saturated and improve the removal efficiency of heavy metals throughout the operation, respectively [18]. In a prototype EKR reactor, which was close to full scale, the electrodes were positioned inside semipermeable electrolyte wells made of polyvinyl chloride cylinders. These electrolyte wells were placed into pre-drilled holes in the soil [19]. However, special precautions must be taken in scaled tests to control the occurrence of potential leaks from the prototype EKR into the surroundings.

Field trial and application methods are based on laboratory-scale designs, but they are susceptible to leakage into the soil. This not only increases the required electrolyte dosage but also carries the risk of secondary soil pollution. In light of these developments, an electrokinetic remediation method that uses electrolytes placed above the ground surface was proposed [20,21]. CA has been used as an effective chelating agent to enhance the removal of heavy metals. However, the electrode arrangements for the EKR of Cu- and Zn-contaminated soft clay and their removal efficiencies using the novel experimental method (electrolyte chamber situated above the soil surface) are not yet clear.

In this context, a series of EKR tests was conducted to investigate how different electrode configurations influence the effectiveness of EKR in heavy-metal-contaminated soft clay. Utilizing a novel electro-remediation experimental setup, this research targets both individual heavy metals (Cu and Zn) and a combined Cu–Zn contamination scenario. Electrokinetic geosynthetics (EKG) served as the electrodes, with 0.2 mol/L citric acid used as the electrolyte solution. This study aims to provide valuable insights for optimizing the design and placement of electrodes to achieve effective electrokinetic remediation of heavy-metal-contaminated soft clay. The findings are intended to guide field applications of electrokinetic remediation, ultimately contributing to the sustainable reuse of polluted soils.

## 2. Materials and Methods

### 2.1. Chemicals

Hydrochloric acid, nitric acid, and perchloric acid reagents used to digest the soil samples were provided by the Analysis and Testing Centre of Nantong University. CuSO_4_·5H_2_O and ZnSO_4_·7H_2_O reagents used to prepare the contaminated soil sample were purchased from Tianjin Zhiyuan Chemical Reagent Company (Tianjin, China). Citric acid was purchased from Sinopharm Chemical Reagent Co., Ltd. (Shanghai, China). All chemicals reached analytical grade.

### 2.2. Simulated Zn and Cu Contaminated Soft Clay

Heavy-metal-contaminated sites are characterized by diversity, complexity, and unevenness, influenced by various factors, making it difficult to analyze the effects of target variables on the removal efficiency of electric remediation for heavy-metal-contaminated soft clay and the migration patterns of pollutants in the soil. This experiment used artificially prepared single heavy metal Cu-, Zn-, and composite heavy metals Cu–Zn-contaminated soft clay as the treatment soil samples.

To prepare soil samples with a target moisture content of 50% and heavy metal concentrations of 500 mg/kg for copper, zinc, and composite copper–zinc contamination, an appropriate amount of clay powder (purchased from Zhangxi Clay Processing Company in Jiangning District, Nanjing, China) with a known initial moisture content was weighed. The required quantities of deionized water, CuSO_4_·5H_2_O, and ZnSO_4_·7H_2_O were then calculated. The basic physicochemical properties of the clay powder are summarized in Table 1. The initial Zn and Cu concentrations in the clay powder were 102 and 0 mg/kg, respectively. 

The masses of deionized water, CuSO_4_·5H_2_O, and ZnSO_4_·7H_2_O reagents that needed to be added were calculated. First, the chemical reagents were added to deionized water and stirred thoroughly until dissolved. Then, the solution was gradually poured into the soil samples, stirring well to ensure even distribution of the pollutants. After sealing the samples, they were placed in a dark environment for one week to simulate conditions similar to real contaminated soil. Next, three contaminated soil samples were taken from different locations, dried in a 105 °C oven for about 10 h, and the actual moisture content was measured. Approximately 0.1 g of each dried soil sample was ground and placed in digestion vessels. We sequentially added 2 mL of HCl, 2 mL of HNO_3_, and 1 mL of HClO_4_, and the mixture was allowed to soak overnight. The next day, the mixture was heated in a fume hood using a heating plate (purchased from Beijing Lebo Tyke Instrument Co., LTD., Beijing, China) set to 200 °C to deacidify and dissolve it. When the mixture color changed from ginger yellow to light yellow and the soil sample turned from brownish-yellow to grayish-white, the digestion was complete. We removed the mixture from the heating plate and allowed it to cool to room temperature. The digested soil sample mixture was diluted with deionized water to a final volume of 40 mL. The solution was filtered through a 0.22 μm syringe filter to remove insoluble residues, yielding 10 mL of the solution to be tested. Finally, we used an atomic absorption spectrophotometer (TAS-990F, manufactured by Beijing Puxi General Instrument Co., Ltd., Beijing, China) to determine the heavy metal content in the solution.

### 2.3. Experimental Setup

**Figure 1:**
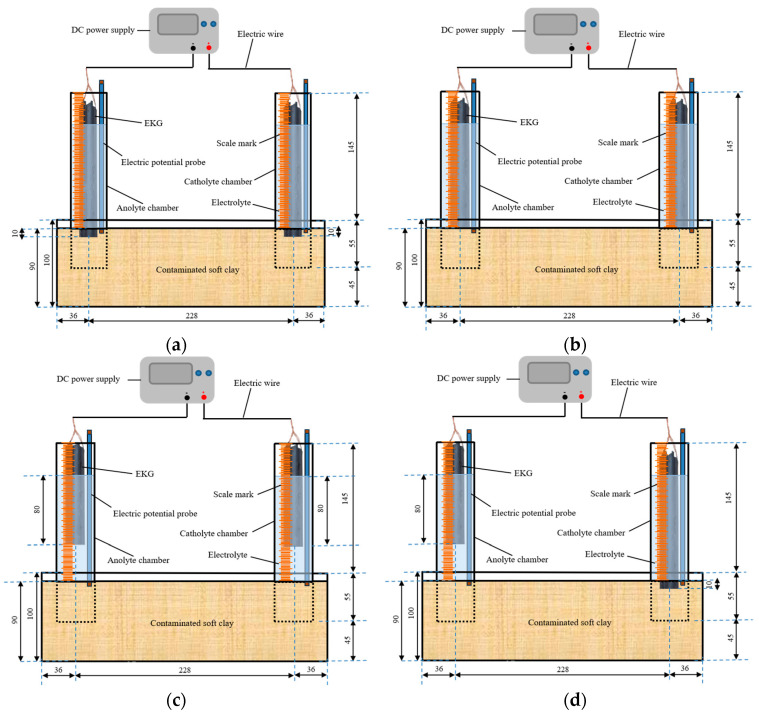

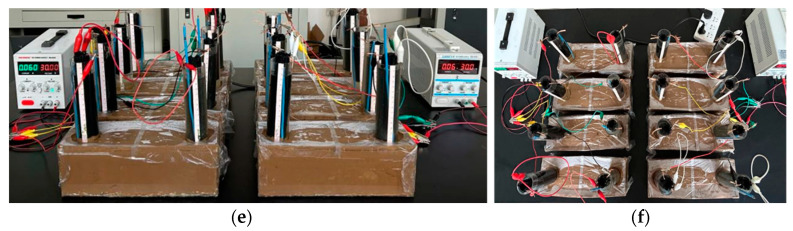
Schematic diagram and realistic scene of the EKR experimental configuration: (**a**) The lower ends of the electrodes in the electrolyte chamber inserted into the soil to 1 cm; (**b**) the lower ends of the electrodes in the electrolyte chamber were in direct contact with the soil surface; (**c**) the length of the anode and cathode in the electrolytes is 8 cm; (**d**) the length of the anode in the anolyte is 8 cm, and the lower end of the cathode in the catholyte chamber is inserted into the soil to 1 cm; (**e**) realistic scene front view; (**f**) realistic scene top view.

The EKR test device used in this experiment is shown in Figure 1. It consisted of a soil sample chamber and two electrolyte chambers placed above the soil sample. The electrolyte and electrodes were placed in the electrolyte chambers, and the electrodes were connected to the DC power supply through wires. The soil sample chamber was made of plexiglass with internal dimensions of 300 mm × 100 mm × 100 mm (length × width × height). The electrolyte chambers were open-ended plexiglass tubes with an outer diameter of 42 mm, an inner diameter of 40 mm, and a length of 200 mm. The outer surfaces of the tubes were marked with a scale. The selected electrodes were tubular and flat-shaped EKGs with good electrical conductivity and corrosion resistance.

These were made of polyethylene, carbon black, and graphite with good conductivity and corrosion resistance [21]. The inner and outer diameters of the tubular EKG were 17 mm and 27 mm, respectively. The flat EKG had a width and a thickness of 40 mm and 6.8 mm, respectively. The electrical conductivity of the flat EKG was inferior to that of the tubular EKG due to the difference in the amount of material used. The direct-current power supply (RXN-605D) had a digital display feature and steady output voltage with a maximum output power of 60 V × 5 A. Rubber-insulated copper electric wires were used to connect the electrodes and the DC power supply.

### 2.4. Test Schemes

Indoor model tests were conducted on soft clay contaminated with copper and zinc, both individually and in combination, using four different electrode placement schemes. These tests aimed to investigate the impact of electrode arrangement on EKR efficiency. The series of EKR tests for soft clay contaminated with copper, zinc, and copper–zinc mixtures were numbered E1a-d, E2a-d, and E3a-d, respectively. The experimental schemes are shown in Table 2. The electrode arrangements were as follows: (a) the bottoms of the electrodes in both the anolyte and catholyte were inserted into the soil by 10 mm, as shown in Figure 1a; (b) the bottoms of the electrodes in both the anolyte and catholyte were in contact with the surface of the soil, as shown in Figure 1b; (c) at the initial moment, both the anode and cathode electrodes had a length of 8 cm in the electrolyte, with their bottoms located 40 mm above the soil surface, as shown in Figure 1c; (d) the electrode in the anolyte had an initial length of 8 cm, with its bottom positioned 40 mm above the soil surface, while the bottom of the electrode in the catholyte was inserted into the soil by 10 mm, as shown in Figure 1d. The actual test setup is shown in Figure 1e,f. Each type of electrode arrangement is described in italics to avoid confusion (i.e., *a*, *b*, *c*, and *d*).

The prepared soft clay contaminated with heavy metals was layered into a soil sample chamber to an initial height of 90 mm. The initial shear strength of the contaminated soil was measured using an indoor electrokinetic vane shear apparatus (TT-LVS, manufactured by Zhejiang Geotechnical Instrument, Shaoxing, China) and determined to be 0.1 kPa. The electrolyte chambers were inserted vertically into the soil to a depth of 45 mm. Subsequently, 120 mL of 0.2 mol/L CA solution was added to both the anolyte and catholyte chambers, which were placed above the soil surface. CA electrolyte enhances the availability of heavy metals in the soil, thereby improving EKR efficiency. Additionally, it is cost-effective and causes minimal changes to soil pollution and properties [3,22]. We inserted an electrode potential probe into each of the anolyte and catholyte chambers, ensuring they contacted the soil at a depth of 1 cm from the surface. These probes monitored the effective electrical potential within the contaminated soil. Each test ran for 10 h daily, with the electrolyte refreshed each day. The voltage was progressively increased from 20 V to 60 V in 10 V increments. The duration of continuous energization for each voltage level in the tests is detailed in Table 2.

Throughout the experiment, the water levels in the anolyte and catholyte were measured hourly to track changes in volume. Given the low permeability of the soil sample (4.0 × 10^−7^ cm/s), the daily permeation loss of the electrolyte solution under the action of gravity can be neglected. Additionally, every 2 h, a multimeter was used to monitor the current and electric potential within the soil. At the end of each day during the experiment, we collected the anolyte and catholyte solutions. We immersed a pH test strip into the solution for 2 to 3 s, then removed it and compared the resulting color with the pH color chart to determine the acidity or alkalinity of the electrolyte solutions.

After the experiment, five cross-sections were taken along the length of the soil sample: one at the center of each electrode chamber (anode and cathode) and three equally spaced sections in between. The water content and shear strength were measured at the top, middle, and bottom of each cross-section. Subsequently, soil samples were collected from the anode, cathode, and intermediate areas using a multi-point equal sampling method. The samples were then dried, ground, and sieved through a 0.15 mm mesh. The concentration of heavy metals in these soil samples was determined using the previously described acid digestion method. Additionally, the dried soil samples were ground and sieved. The soil powder was combined with deionized water at a soil-to-water ratio of 1:5 and mixed thoroughly. The mixture was allowed to settle for 30 min. Then the supernatant was collected, and its pH was measured using pH test paper (purchased from Shanghai SSS Reagent Co., Ltd., Shanghai, China). To assess the reproducibility of these procedures, duplicate experiments were conducted. Due to the close similarity of the results, only one set of experimental results is presented here, along with the associated error. In addition, in this study, the accuracy of the amount of contaminated soil sample materials configured was ensured, and the uniformity of heavy metals in the soil was improved as much as possible. The heavy metal concentration was measured at multiple different points for the configured soil sample to ensure that the error was below ± 20 mg/kg. For the soil sample after EKR treatment, a multi-point equal sampling method was used, and the concentration distribution of heavy metals in different sections was tested with parallel samples to ensure the accuracy of the test results.

## 3. Results and Discussion

### 3.1. Electric Current

**Figure 2:**
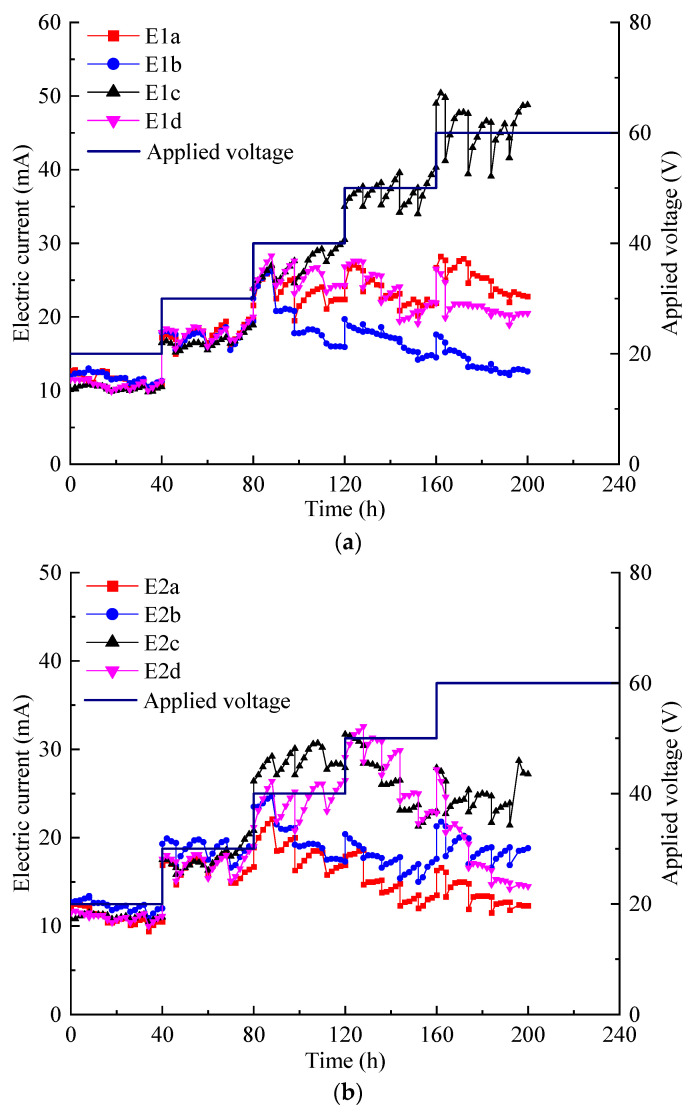

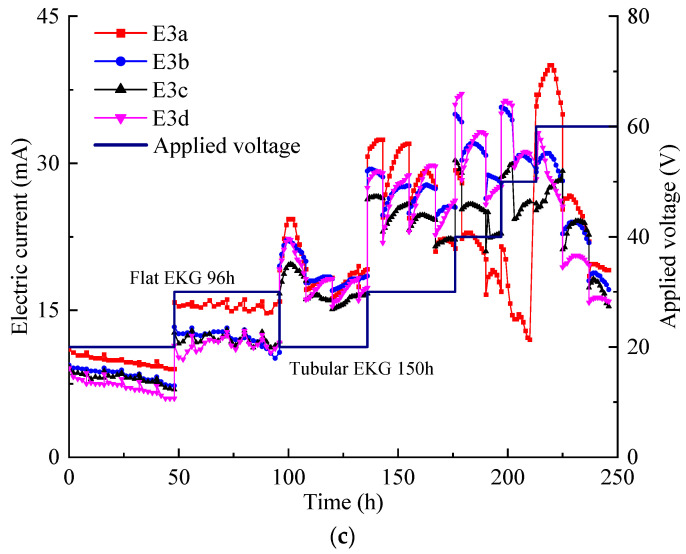
Electric current variation: (**a**) E1*a*–E1*d*; (**b**) E2*a*–E2*d*; (**c**) E3*a*–E3*d*.

The current variation in Cu-contaminated soft clay is depicted in Figure 2a. At voltages of 20 and 30 V, the currents in tests E1*a*–E1*d* were similar. However, as the voltage increased to 40 V, the current in E1*c* steadily rose, and at voltages of 50 and 60 V, it became significantly higher than in the other tests. Conversely, under higher voltages, the current in E1*b* decreased gradually and was lower than in the other tests. The current reflects the chemistry of the pore solution. The increased desorption of heavy metals and other ions from soil particles and their entry into the pore fluid led to a decrease in the electrical resistance of the soil [18].

The current variation in Zn-contaminated soft clay, as depicted in Figure 2b, shows minimal differences among E2*a*–E2*d* at voltages of 20 and 30 V. However, at voltages between 40 and 60 V, the current in E2*c* and E2*d* exhibited an initial increase followed by a decrease, while E2*a* and E2*b* maintained relatively stable currents. By the end of the experiment, E2*c* recorded the highest current, followed by E2*b*, E2*d*, and E2*a* in descending order. The current in each test at all voltage levels was almost always below 30 mA. The electric current is considered a prominent factor influencing the extraction efficiency for heavy metals [23,24]. However, the current in each experiment at various voltage levels was consistently below 30 mA, which is insufficient for total heavy metal removal.

The current variation in soft clay contaminated with Cu and Zn, as depicted in Figure 2c, was as follows: During the first 96 h, when a flat EKG was used, the current remained relatively low in all experiments under 20 and 30 V, with E3*a* showing slightly higher current than the other tests. After switching to tubular electrodes, the current significantly increased under 20 and 30 V in all experiments, but there were substantial fluctuations, especially between 30 and 60 V. At 40 and 50 V, the current in E3*a* gradually decreased, and at 60 V, a rapid decline in current was observed across all experiments.

According to Ohm’s Law, for a given voltage, the current in a circuit is determined by its resistance. At lower voltages of 20 and 30 V, the electrode configuration had minimal impact on circuit resistance. However, when the voltage exceeded 40 V, electrode arrangement *c* became more effective at reducing circuit resistance, which in turn enhanced the current flow. This method of electrification introduced more acidic anolytes into the soil, leading to the desorption of some heavy metals from the surface of the soil particles and their dissolution into the pore water. The current intensity is strongly related to the concentration of mobile ions in the soil [25].

Even with identical electrode arrangements and the same applied voltage, the current in Cu-contaminated soft clay differed from that in Zn-contaminated soft clay. At voltages of 40–60 V with electrode configuration mode *a*, and at voltages of 50–60 V with electrode configuration mode *c*, the current in copper-contaminated soft clay was notably higher compared to zinc-contaminated soft clay. The rate at which current decreased in soft clay contaminated with both Cu and Zn at voltages above 40 V was greater than in soft clay contaminated with either Cu or Zn alone. During the EKR process, the volume of pore fluid in the soil and the concentration of mobile ions within it gradually decreased [26]. Simultaneously, the formation of cracks in the soil led to a continuous increase in soil resistance. Consequently, the current declined in the later stages of the experiment.

### 3.2. Contaminated Soil Resistance Variation

As shown in Figure 1, the electric potential of the soil surface inside the anolyte and catholyte chamber was tested by the electric potential probes in the E1 and E2 series tests. The effective electric potential in the soil ($U_{e}$) is defined as the electric potential of the soil surface inside the anolyte chamber minus the electric potential of the soil surface inside the catholyte chamber [20]. Using the contaminated soil resistance ($R_{s}$), based on the current (I) in the circuit, it can be roughly concluded that:

(1) $$R_{s}=\frac{U_{e}}{I}$$

**Figure 3:**
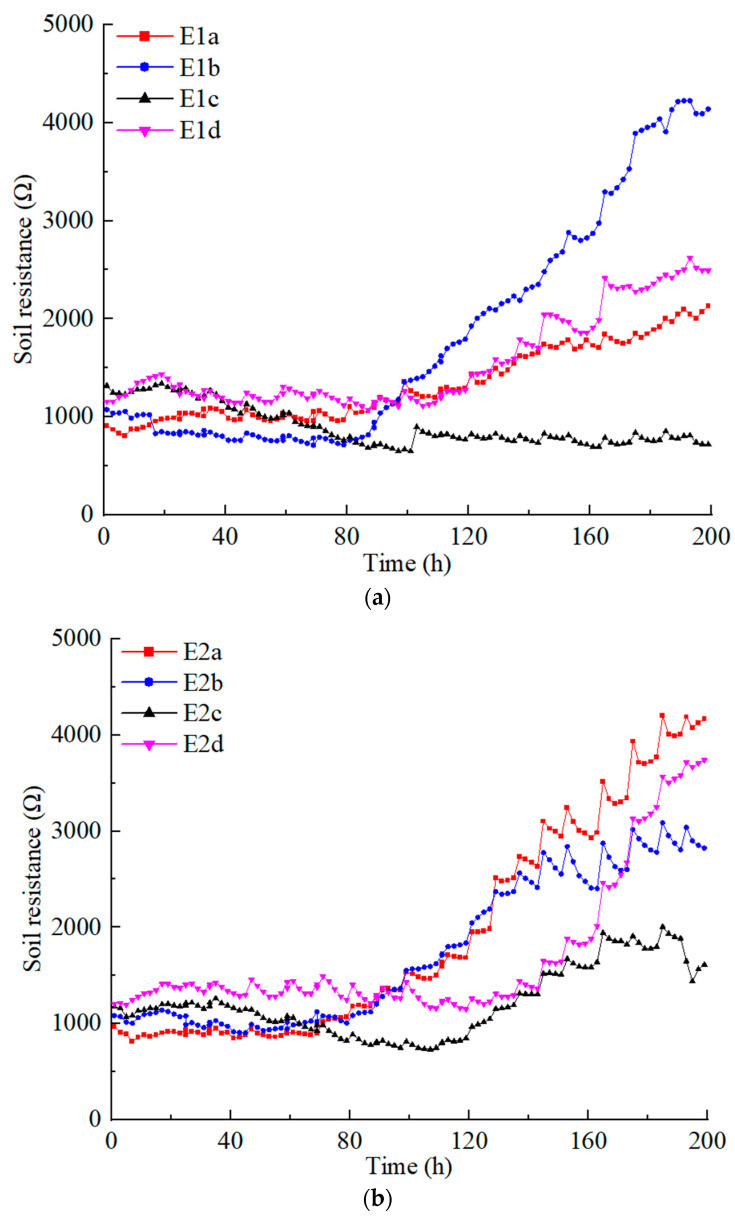
Contaminated soil resistance variation: (**a**) E1*a*–E1*d*; (**b**) E2*a*–E2*d*.

Figure 3 illustrates the variations of the contaminated soil resistance in the E1 and E2 series tests under different applied voltages. The position of the electrodes significantly affects the resistivity of soft clay contaminated individually by copper and zinc. During the first 80 h, under low voltage conditions (20 and 30 V), the soil resistivities in the E1 and E2 series tests were relatively similar and exhibited minimal variation. Notably, the soil resistivity with electrode arrangement *d* was somewhat higher compared to other configurations, whereas with electrode arrangement *c*, there was a noticeable decreasing trend in soil resistivity. When the voltage exceeded 40 V, the resistivity of contaminated soil showed differences depending on the arrangement of the electrodes. When copper-contaminated soft clay was subjected to electrode arrangement *b*, the soil resistivity increased rapidly and reached its maximum value at voltages of 40 V and above. For electrode arrangements *a* and *d*, the soil resistivity showed an increasing trend once the voltage reached 50 V. In contrast, under electrode arrangement *c*, the soil resistivity consistently remained at a relatively low level. In zinc-contaminated soft clay, the soil resistivity increased rapidly at voltages of 40 V and above with electrode arrangements *a* and *b*, with the highest resistivity recorded for arrangement *a*. For electrode arrangements *c* and *d*, the resistivity began to rise when the voltage reached 50 V. At 60 V, the increase in resistivity was rapid for arrangement *d*, whereas for arrangement *c*, the resistivity increase began to stabilize.

Under the same voltage and electrode arrangements, differences were observed in the resistivity of soft clay contaminated by copper and zinc. At low voltage, these differences were relatively minor. However, at high voltage, with electrode arrangements *a*, *c*, and *d*, the resistivity of zinc-contaminated soft clay was significantly higher than that of copper-contaminated soft clay. In contrast, with electrode arrangement *b*, the resistivity of zinc-contaminated soft clay was markedly lower than that of copper-contaminated soft clay.

### 3.3. Phenomena and pH in Electrolytes

The CA concentrations of the anolytes and catholytes in the three series tests were 0.2 mol/L, with initial pH values of 2.5. During EKR treatment, the electrolytes were refreshed one time per day (daily working hours were 10 h). The pH values of the replaced anolytes and catholytes were tested in time. After each test, the daily test results of the pH values of all the replaced electrolytes were analyzed. The anolyte pH value of each test basically dropped to about 2.0 each time, while their catholyte pH values remained unchanged at 2.5. The electrolytes were always kept in an acidic environment with a low pH. Hence, the CA solution effectively controlled the pH of the anolyte and catholyte during EKR treatment. The pH distribution in the soil was consistent across all tests after EKR treatment: the pH value was 4.0 in the anode section S1, 5.0 in the S3 section, and 5.5 in the cathode section S5. pH value has a significant impact on the desorption of heavy metals from the surface of the soil particles into the liquid phase and the subsequent migration [27,28].

During EKR treatment, the anode surface mainly undergoes electrolytic water molecule reactions to generate oxygen and hydrogen ions, resulting in a decrease in pH value. The pH of the anolyte dropped from an initial value of 2.5 to 2. In each experiment, the anolyte turned pale yellow, and bubbles formed on the surface of the anode. The chemical reaction equation is as follows:

2H_2_O − 4e^−^→ O_2_ ↑ + 4H^+^ (2)

Under the influence of an electric field, the types and quantities of cations migrating to the catholyte were affected by the position of the electrodes. The substances precipitated at the cathode mainly depended on the standard reduction potential of the cations and their concentration. According to the Nernst equation [29]:

(3) $$E=E^{0}-\frac{RT}{zF}lnQ$$

where E is the cell potential under non-standard conditions, $E^{0}$ is the standard cell potential, R is the ideal gas constant, T in temperature in Kelvins, z is the stoichiometric number of moles of electrons transferred, F is the Faraday constant, and Q is the reaction quotient (the ratio of the concentrations of the products to the reactants). High concentrations of reduced species led to an increase in electrode potential, making reduction reactions more likely to occur. Since the pH of the catholyte was consistently maintained at 2.5, the concentration of H^+^ remained relatively high. Typically, Cu^2^⁺ or H^+^ was preferentially reduced, resulting in the deposition of metallic copper or the evolution of hydrogen gas at the cathode. Bubbles were observed on the surface of the cathode in all experiments, and the chemical reaction equations were as follows:

Cu^2^⁺ + 2e^−^ → Cu (4)

2H^+^ + 2e^−^ →H_2_ ↑ (5)

In this experiment, the electrolyte was placed on top of the soil, and the electrodes were vertically inserted into it at varying depths. This approach to applying an electric current led to the convergence of more electric field lines at the bottom of the electrodes due to edge effects, resulting in relatively higher electric field intensity and charge density in that region. In tests where the cathode was buried 1 cm in the soil (E1*a* and E1*d*, E2*a* and E2*d*, E3*a* and E3*d*), white precipitates were observed forming at the bottom of the cathode. The high electric field strength and charge density at the cathode base led to the electrolysis of water, producing OH^−^ ions. These ions, along with cations in the soil (such as Ca^2^⁺, Al^3^⁺, and Mg^2^⁺), migrated towards the cathode under the electric field. This process resulted in the precipitation of white hydroxides, which adhered to the cathode base and diminished the electrode reactivity.

### 3.4. Electroosmotic Flow and Electrophoresis

**Figure 4:**
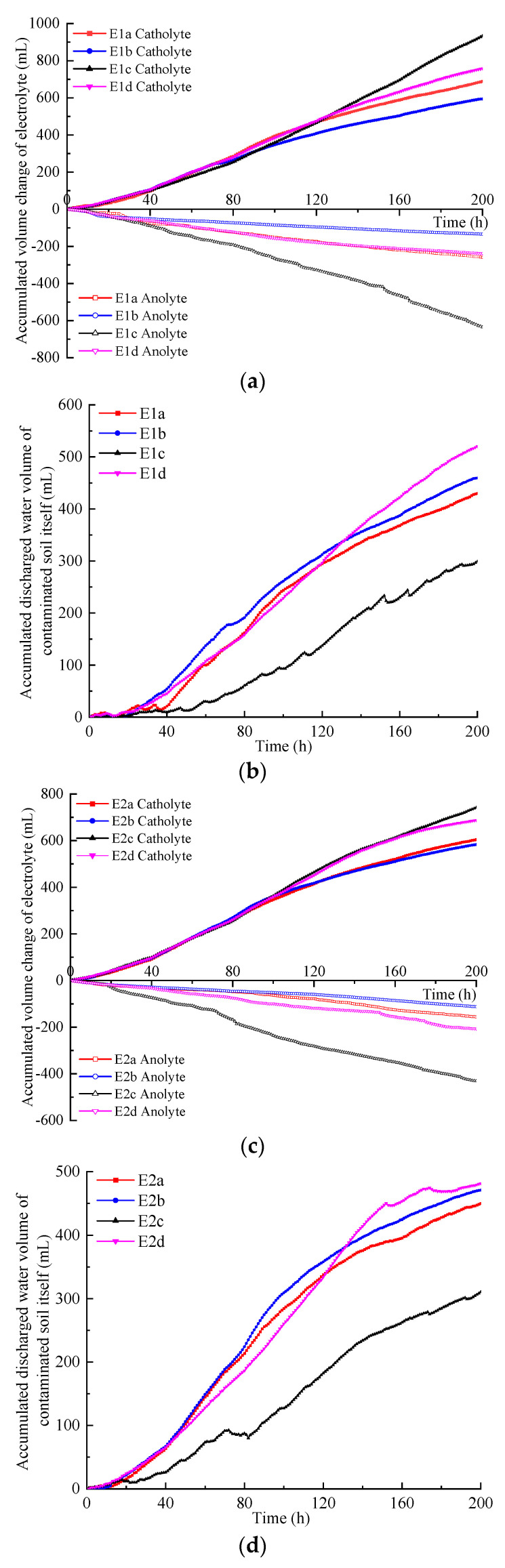

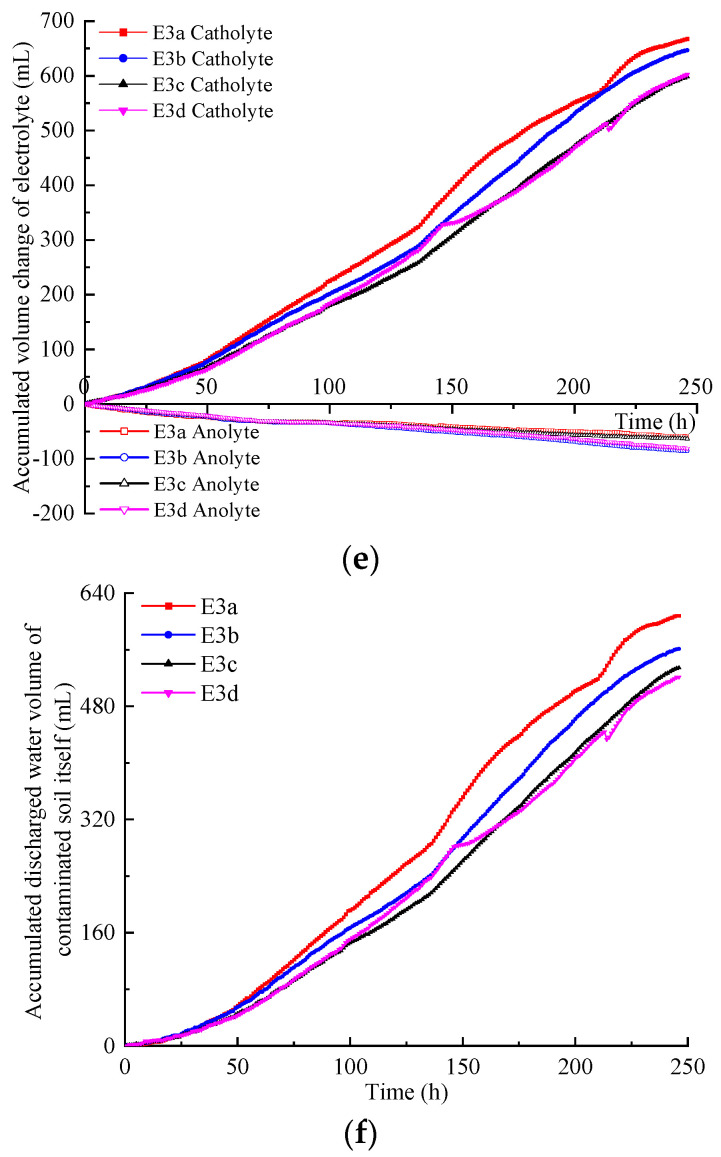
Electroosmotic flow: (**a**) Accumulated volume change in the electrolyte in the E1 series tests; (**b**) accumulated discharged water volume of the contaminated soil in the E1 series tests; (**c**) accumulated volume change in the electrolyte in the E2 series tests; (**d**) accumulated discharged water volume of the contaminated soil in the E2 series tests; (**e**) accumulated volume change in the electrolyte in the E3 series tests; (**f**) accumulated discharged water volume of the contaminated soil in the E3 series tests.

Under a direct current electric field, hydrated cations in the anolyte and contaminated soil migrate toward the cathode, generating electroosmotic flow. Consequently, the volume of the anolyte decreases, while the volume of the catholyte increases. The difference between the accumulated volume increase in the catholyte and the accumulated volume decrease in the anolyte at the same time is the accumulated volume of discharged water of the contaminated soil. The electroosmotic flow observed in the anolyte, catholyte, and contaminated soil for each experiment is illustrated in Figure 4. It can be observed that, in each test, the volume of catholyte accumulated increased nearly linearly, while the volume of anolyte decreased nearly linearly.

For the E1 series tests, the volume increase in the catholyte showed differences under 40 V, with mode *c* having the largest volume increase, followed by modes *d*, *a*, and *b*. For the anolyte, the volume decrease showed differences under 30 V, with mode *c* having the largest volume decrease. Modes *a* and *d* consistently had the same volume decrease, while mode *b* had the smallest volume decrease. The increase in catholyte volume was consistently greater than the decrease in anolyte volume across all modes. Copper-contaminated soil began draining at approximately 30 V. At 30 and 40 V, the drainage volume of the soil in modes *a*, *b*, and *d* was relatively similar, whereas at 50 and 60 V, mode *d* exhibited higher drainage compared to modes *a* and *b*. In all instances, the drainage volume of the soil in modes *a*, *b*, and *d* was consistently higher than that in mode *c*.

For the E2 series tests, the increase in the volume of catholyte in modes *a* and *b*, as well as in modes *c* and *d*, remained consistently close. The decrease in the volume of anolyte in mode *c* was sequentially higher than in modes *d*, *a*, and *b*. The zinc-contaminated soft clay began to drain at a voltage of 20 V, with the drainage amounts in modes *a*, *b*, and *d* being consistently similar and greater than in mode *c*. Under the same electrode arrangement pattern, the volume changes of the electrolyte in both the anode and cathode for zinc-contaminated soft clay were lower than those for copper-contaminated soft clay. However, the net drainage volumes of the two types of contaminated soils were relatively similar. The maximum drainage occurred in mode *d*, while mode *c* exhibited the minimum drainage for both E1 and E2 series tests.

The volume reduction in the anolyte in E3 series tests remained consistently low, indicating minimal change. In contrast, the volume increase in the catholyte significantly surpassed that of the anolyte, suggesting that the increase in catholyte volume primarily originated from contaminated soil. The volume increases in the catholyte in modes *c* and *d* were similar, though slightly lower than in modes *a* and *b*. Mode *a* showed the highest soil drainage volume, while modes *c* and *d* exhibited similar and the lowest soil drainage volumes.

In the E1 and E2 series tests, when the electrode position was in mode *c* (electrodes suspended in the electrolytes), the volume reduction in the anolyte was the greatest, and the soil drainage volume was the lowest. When the electrode position was in mode *b*, the volume reduction in the anolyte was the smallest. When the electrode position was in mode *d*, the soil drainage volume was the greatest.

**Figure 5:**
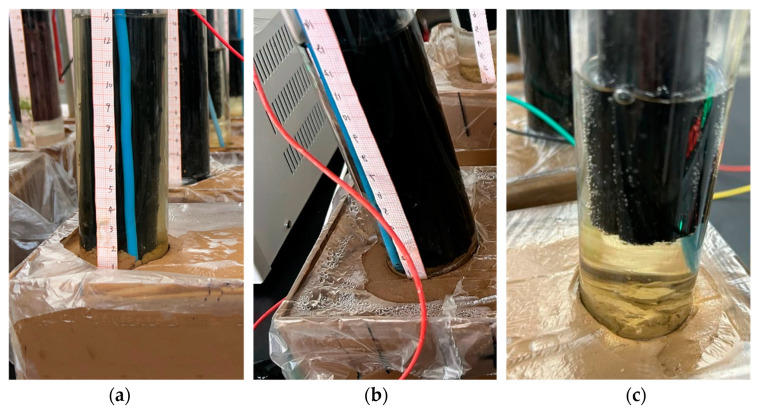
Electrophoresis phenomenon in the anode chamber: (**a**) Mode *a*; (**b**) Mode *b*; (**c**) Mode *c* and *d*.

In the E1, E2, and E3 series of tests, electrophoresis occurred at the bottom of the anode tube, and the timing of the electrophoresis phenomenon generally aligned with the pattern described by Sun et al. [30]. There is a threshold current (about 15 mA) for electroosmosis and electrophoresis in soft clay. Below the threshold current, electrophoresis plays a major role and is accompanied by weak electroosmosis. Above the threshold current, almost only electroosmosis occurs. The E1 and E2 series tests generally exhibited the phenomenon of soil particles rising in the anode chamber under a voltage of 20 V for the first 40 h. The E3 series tests, during the use of flat EKG, also demonstrated the rise of soil particles in the anode chamber under voltages of 20 and 30 V for the first 96 h, with currents less than 15 mA. However, the speed and height at which the electrophoresis soil particles rose in the anode chamber were very limited. In mode *a*, the electrophoresis soil extended 1–2 cm above the original soil surface, while in modes *b*–*d*, it extended 0.5–1 cm above the original soil surface, as shown in Figure 5. On one hand, this was due to the pH of the anolyte being 2.0, which contained a high concentration of hydrogen ions, resulting in the zeta potential of the soil particles in the anode chamber becoming less negative [31]. On the other hand, this was due to the decomposition of aluminosilicates in the soil, which produced silicate ions. These ions reacted with Ca^2+^ in the soil, leading to the formation of calcium silicate hydrate, causing the soil particles to cement together into clumps. The chemical reaction was as follows:

Ca^2+^ + SiO_3_^2−^ + H_2_O → CaSiO_3_ ∙ H_2_O (6)

The soil rose higher in mode *a* electrophoresis compared to modes *b* and *c* because the electrode in mode *a* was inserted 1 cm deep into the soil. The bottom of the annular electrode with the highest surface curvature had the highest amount of charge, resulting in a strong electrolysis reaction of water to generate gas. Fine soil particles with an upwelling height of 5–6 mm, accompanied by rising bubbles, were observed in both the anode and cathode chambers. This suggests that the upwelling of soil was caused by the vigorous rise of bubbles generated at the bottoms of the electrodes rather than electrophoresis.

### 3.5. Water Content and Shear Strength

**Figure 6:**
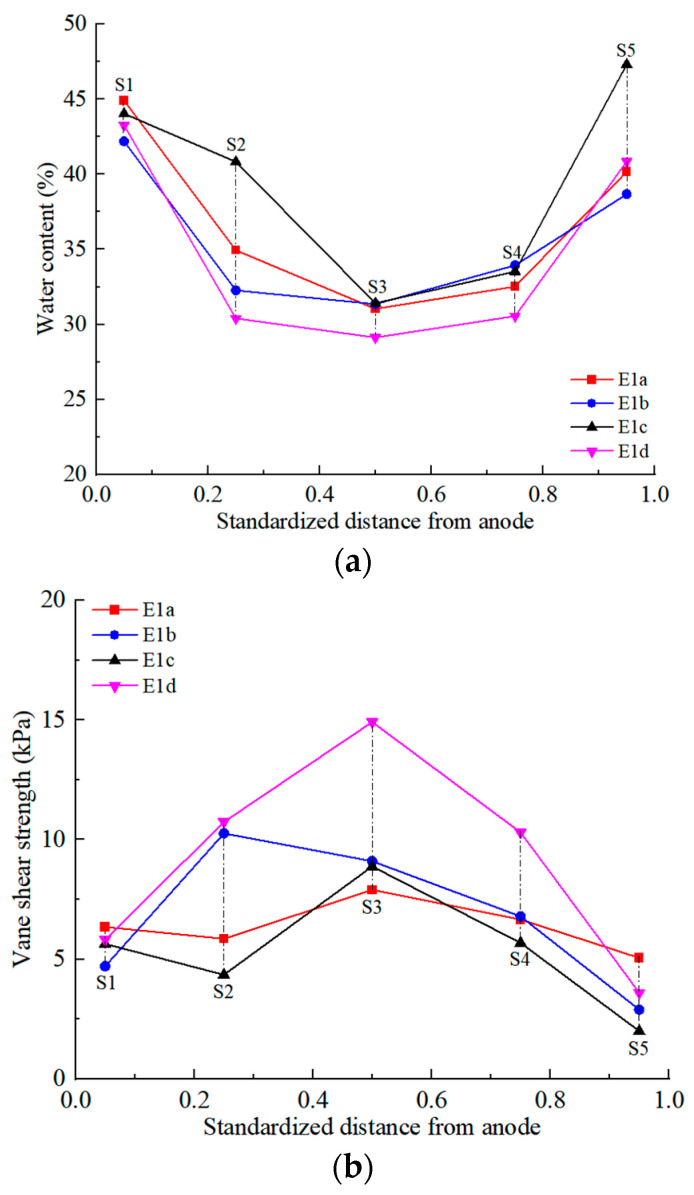

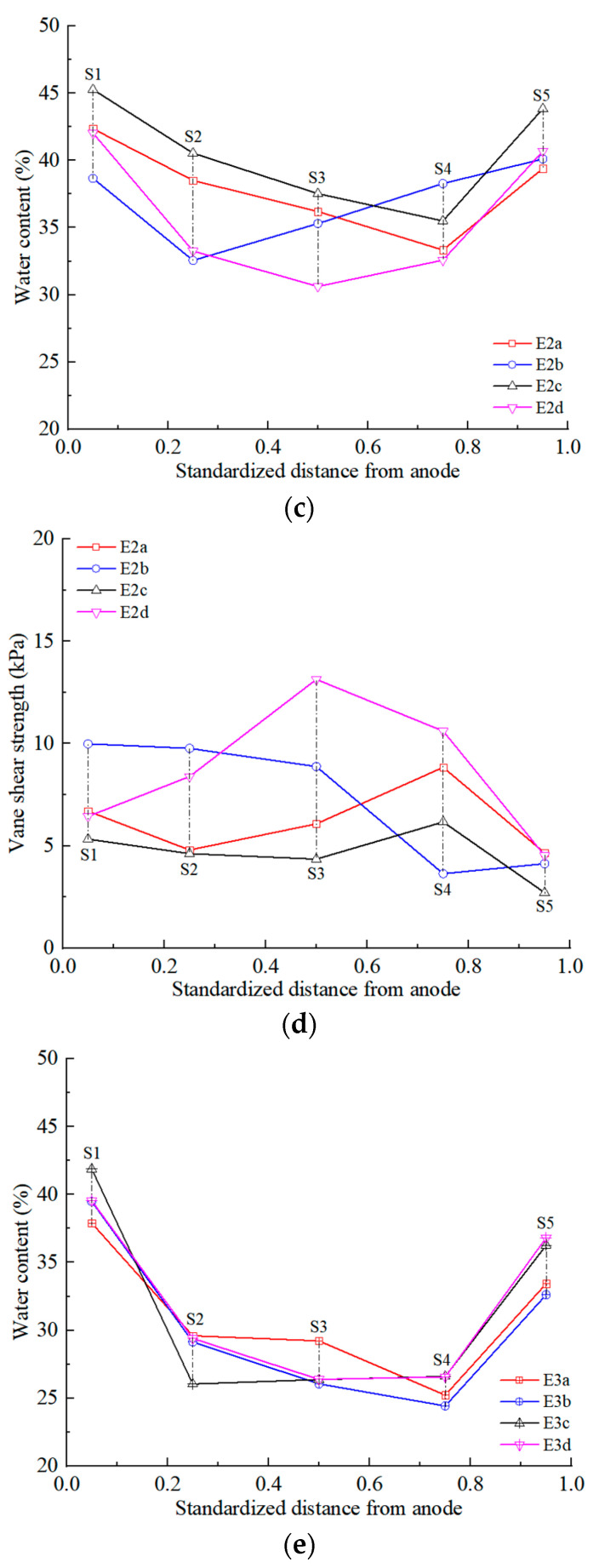

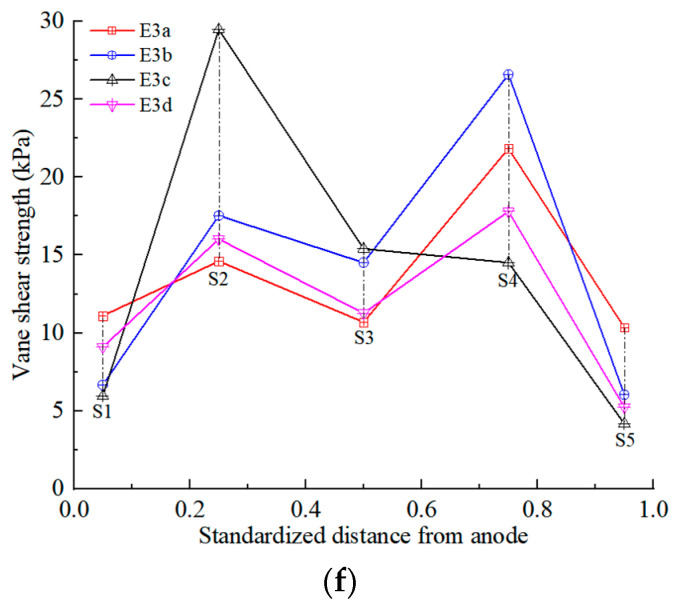
Water content and vane shear strength distribution in the soil after EKR treatment: (**a**) water content distribution in E1 series tests; (**b**) vane shear strength distribution in E1 series tests; (**c**) water content distribution in E2 series tests; (**d**) vane shear strength distribution in E2 series tests; (**e**) water content distribution in E3 series tests; (**f**) vane shear strength distribution in E3 series tests.

After EKR treatment, soil water content was tested according to the testing points layout diagram in Figure 1c. The testing points were distributed at the surface, middle, and bottom positions of five cross-sections (S1–S5). The average moisture content of each section was calculated, and the distributions are shown in Figure 6. Shear strength is an important mechanical property of soil. After EKR treatment, the shear strength of the soil was firstly tested according to the testing points layout diagram in Figure 1c. The average shear strength at each section was calculated, and their distributions are shown in Figure 6.

In the E1 series of tests, the moisture content was lowest at the middle cross-section S3 between the anode and cathode. The moisture content increased as one moved from this cross-section toward the anode and cathode. The vane shear strength of soil in S3–S5 sections decreased with increasing moisture content.

The shear strength of the soil at the S1–S3 cross-section was influenced by the electrode arrangement. In electrode arrangement modes *a* and *c*, the water content of the soil near the anode at the S1 cross-section was higher than at the S2 cross-section. However, due to chemical cementation in this area, the shear strength of the soil at the S1 cross-section was slightly higher than at the S2 cross-section. Under the *b* electrode mode, the maximum shear strength occurred in section S2. In the *d* electrode mode, the moisture content at the middle section S3 was not significantly different from that in adjacent sections S2 and S4, but the shear strength in section S3 was 5 kPa higher than in sections S2 and S4.

In the E2 series tests, the moisture content in section S4 was the lowest, and the shear strength was the highest under electrode configurations *a* and *c*. Although there were variations in moisture content in sections S1 to S3, their shear strengths were relatively similar. Under electrode configuration *b*, the moisture content in section S2 was the lowest, whereas the shear strengths in sections S1 to S3 were close to each other and higher than those in sections S4 and S5. The distribution patterns of moisture content and shear strength in E2*d* were similar to those in E1*d*.

In the E3 series tests, the moisture content was higher near the electrodes in sections S1 and S5, while the moisture content in sections S2 to S4 was similar. In electrode arrangement patterns *a*, *b*, and *d*, there was a sudden increase in shear strength in sections S2 and S4, with the highest shear strength observed in section S4. In the electrode arrangement pattern *c*, the maximum shear strength was found in section S2, where the moisture content was the lowest at approximately 25%, and the shear strength reached about 30 kPa, which was higher than in the other series of tests.

The water content of the soil in the E1 and E2 series tests typically ranged from 30% to 45%, with a shear strength between 2 and 15 kPa. In the E3 series tests, the soil water content ranged from 25% to 42%, and the shear strength ranged from 2 to 30 kPa. High water content could enlarge the pore size and shorten the flow and ion path during EKR, which indicates that water content could enhance the overall removal of heavy metals in EKR [13]. The moisture content distribution of the soil in each experiment was related to the drainage volume of the soil during the EKR process.

In the E3 series tests, the contaminated soil led to a higher net drainage volume, which in turn reduced the water content relative to E1 and E2, thus increasing the shear strength. The shear strength of the contaminated soil sample was nearly zero before treatment. Following EKR, there was a modest increase in the shear strength of the contaminated soft clay across various sections. This suggests that EKR can effectively improve soil strength and the mechanical properties of contaminated soft clay, although the overall enhancement is relatively modest.

### 3.6. Heavy Metal Concentration

**Figure 7:**
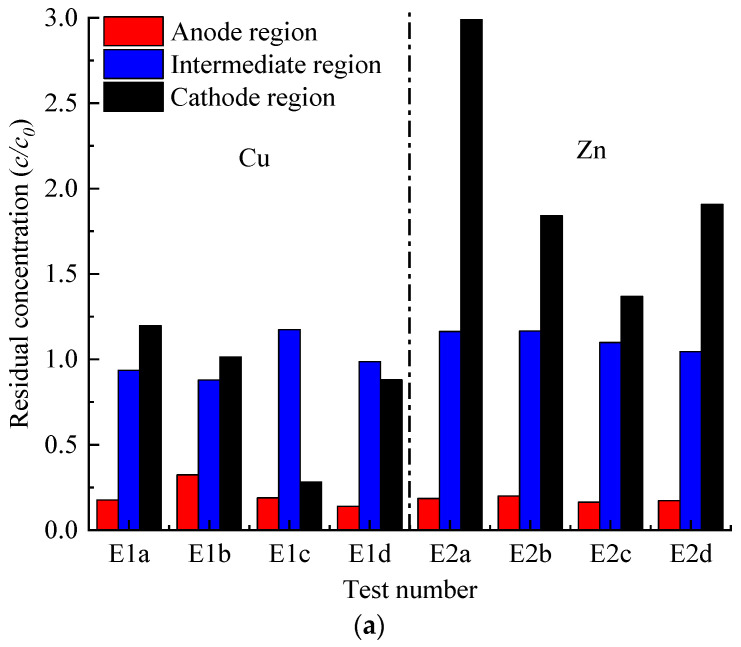

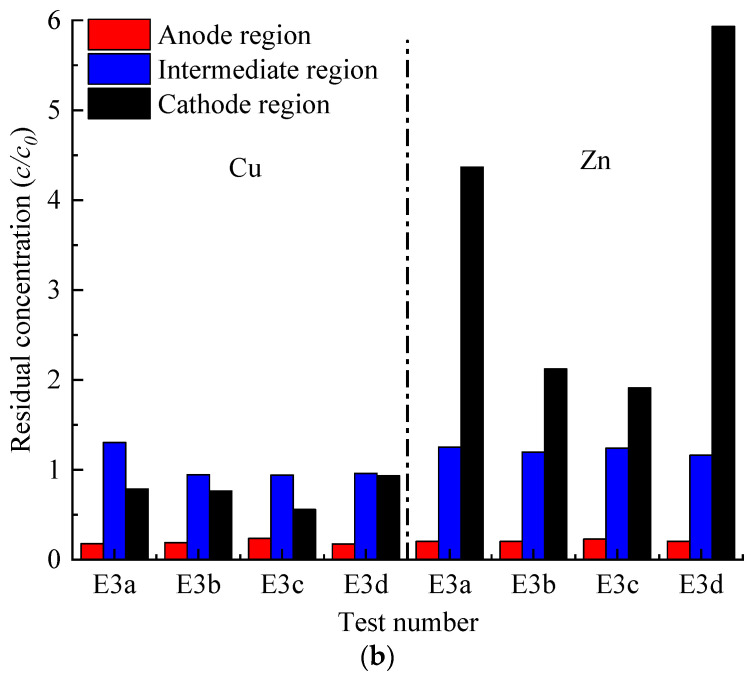
Heavy metal distribution in sediment after EKR treatment: (**a**) Residual concentration of E1 and E2 series tests; (**b**) residual concentration of E3 series tests.

The distributions of heavy metals in the soil section after EKR treatment are illustrated in Figure 7. The removal efficiencies of Cu and Zn were best in the anodic areas in each test. The differential removal of Cu and Zn was observed in the middle area of the specimen and the cathodic area. However, Zn accumulated in both the middle area of the specimen and the cathodic area in the E2 and E3 series tests, with a particularly severe accumulation observed in the cathodic area. When the cathode was buried 1 cm into the soil (modes *a* and *d*), the accumulation of zinc in the cathode region was the most pronounced. This is followed by the cathode being in contact with the soil (mode *b*). The least accumulation occurred when the cathode was suspended in the electrolyte without contacting the soil.

According to the heavy metal concentrations measured in the digested soil samples, the heavy metal removal rates (η) in the three parts of each test were calculated according to the following equation:

(7) $$\eta =\frac{c_{0}-c}{c_{0}}\times 100\%$$

where *c*_0_ (mg/kg) is the initial heavy metal concentration in the polluted soil, and *c* (mg/kg) is the heavy metal concentration of the soil after EKR treatment. In some instances, there was even a negative removal of target metals due to the accumulation generated at certain locations during the heavy metal migration process, which leads to the concentration of heavy metals after EKR treatment exceeding the initial concentration [23,32,33].

In the E1 series of experiments, copper removal in the middle region was slightly enhanced in modes *a*, *b*, and *d*, while copper accumulation occurred in mode *c*. In the cathode region, mode *c* exhibited the highest copper removal rate at 71.8%, while mode *d* showed a removal rate of 12.0%. Conversely, copper accumulation was observed in modes *a* and *b*. In the E3 series of experiments, copper removal in the middle region was slightly enhanced in modes *b*, *c*, and *d*, while it accumulated in mode *a*. In the cathode region, mode *c* exhibited the highest copper removal rate at 44.2%, while modes *a* and *b* showed close removal rates of 21.4% and 23.6%, respectively. The removal rate was slightly enhanced in mode *d*.

In previous studies by Sun et al. (2023) [21] and Sun et al. (2024) [20], the same apparatus (mode *c*) and CA as the electrolyte were used to conduct EKR experiments on soft clay contaminated with heavy metal zinc and a mixture of copper and zinc, respectively. In the study by Sun et al. (2023) [21], the concentration of heavy metal zinc pollution was 3175.4 mg/kg. Under a stable potential gradient of 1.5 V/cm, with the 0.2 M CA anolyte and catholyte being renewed daily, after 48 days of treatment (9 h per day, totaling 432 h), the removal efficiency across different soil sections was relatively uniform, and more than 90% of the initial Zn was removed. In the study by Sun et al. (2024) [20], the contamination concentrations of heavy metals copper and zinc were both 500 mg/kg. Under a stepwise voltage increase from 20 to 60 V and daily replacement of 120 mL of 0.2 M CA anolyte and catholyte, after 260 h of treatment, the removal rates of zinc in the anode region, middle region, and cathode region were 89.0%, 55.7%, and 46.9%, respectively, while the removal rates of copper were 78%, 36%, and −30%, respectively.

Compared to Sun et al. (2023) [21], the experimental conditions of E2*c* differed in two main aspects: (1) the initial concentration of the heavy metal Zn in E2*c* was much lower than that reported in the literature; (2) the EKR time of E2*c* was significantly shorter than that in the literature. The removal rate of heavy metal zinc increases with higher initial concentrations and longer remediation times [17,23]. These factors resulted in a better removal rate of 83.6% for E2*c* in the anode region, while accumulation occurred in the cathode and middle regions, with removal rates of −9.9% and −36.8%, respectively.

The experimental conditions of E3*c* differed from those of Sun et al. (2024) [20] in two main aspects: (1) E3*c* used flat EKGs during the initial 96 h; (2) the EKR time in E3*c* was slightly shorter. In the E3c process, the removal rates of zinc in the anode region, middle region, and cathode region were 77.0%, −24.2%, and −91.1%, respectively. For copper, the removal rates were 76.4%, 5.8%, and 44.2%, respectively. The zinc removal rates in the middle and cathode regions were significantly lower than those reported by Sun et al. (2024). Conversely, the copper removal rate in the cathode region was better than that reported by Sun et al. (2024) [20], although the removal rate in the middle region was poorer. The first point of difference resulted in a decrease in the volume of E3*c* anolyte, while the volume of contaminated soil drainage was relatively high. This led to insufficient extraction of heavy metals from soil particles and had a more significant impact on the removal rate of the heavy metal zinc.

Furthermore, it should be noted that, compared to Sun et al. (2023) [21] and Sun et al. (2024) [20], the currents in the E1, E2, and E3 series of experiments were relatively low, while higher currents facilitated the desorption and movement of more heavy metal ions [23].

By comparing the average removal rates of copper and zinc in the E1, E2, and E3 series tests, as presented in Table 3, it is evident that the removal efficiency for copper (Cu) surpasses that for zinc (Zn). The stability constant of CA with Cu was higher than that with Zn. Complexation of citrate–metal enhanced the removal efficiency and transfer of heavy metals [18]. Additionally, copper ions have a smaller radius compared to zinc ions. A smaller cationic radius and higher electric charge contribute to an increased ionic mobility [28].

### 3.7. Discussion

Using this experimental setup, due to boundary effects, there was a higher accumulation of charge and a stronger electric field in the lower-end region of the electrode. As a result, the position of the electrode end had a significant impact on the migration of ions in the anolyte and catholyte, which in turn affected the volume reduction in the anolyte, the magnitude of the current, and the migration of heavy metals, as shown in Table 3. In this experiment, the reduction in the anolyte was primarily due to the migration of hydronium ions (H₃O^+^) towards the cathode. This process, on the one hand, directly increased the number of migrating ions, thereby increasing the amount of charge passing through per unit of time, which resulted in a higher current. On the other hand, the migration of hydronium ions into the contaminated soil lowered the soil pH, causing some heavy metals to desorb from soil particles. This led to an increase in the concentration of free heavy metals in the pore fluid, further enhancing ion migration. Therefore, the cumulative reduction in the volume of the anolyte had a significant impact on the removal rate of heavy metals.

When the cathode and anode were suspended in the electrolyte, with the lower ends of the electrodes kept at a certain distance from the surface of the soil (in *c* mode), the ions in the electrolyte between the lower ends of the electrodes and the soil surface experienced a stronger electric field force. This increased the number of migrating ions and resulted in an increase in current. The hydrated hydrogen ions in the anolyte exhibited higher mobility compared to other ions in the system, facilitating rapid movement through the anolyte [25]. In the E1 and E2 series tests, the cumulative volume reduction in the anolyte under mode *c* was significantly higher than in other modes, yet the net drainage volume of the contaminated soil was the smallest. When the anode was suspended in the electrolyte and the end of the cathode was inserted 1 cm deep into the soil (mode *d*), the number of anions migrating from the catholyte towards the anode was reduced. Under the same voltage, the current (electric field strength) decreased compared to mode *c*, which resulted in a significantly reduced cumulative volume of the anolyte. This also promoted the maximum drainage of soil in this mode. However, this reduction actually results in the highest drainage volume in the soil under this mode.

In the E3 series of experiments, it was also observed that the reduction in the volume of the anolyte was minimal in each test, while the amount of water drained from the soil significantly increased. This is because the large influx of hydrogen ions from the anolyte into the contaminated soil weakened the normal electroosmotic flow rate by reducing the absolute value of the zeta potential of the soil particles [31]. When the amount of anolyte entering the contaminated soil was relatively small, the impact on the electroosmotic flow rate in the contaminated soil was not significant, but the amount of drainage from the soil increased significantly. The electroosmotic flow rate ($q_{eo}$) is given by [34]:

(8) $$q_{eo}=k_{eo}EA$$

(9) $$k_{eo}=\frac{\varepsilon \varepsilon_{0}\zeta }{\eta }n_{e}\tau$$

where $q_{eo}$ is electroosmotic flow rate; $k_{eo}$ is electroosmotic permeability; E is the field strength; A is cross-sectional area; ε is the relative permittivity of the pore fluid; $\varepsilon_{0}$ is the permittivity of free space; ζ is the zeta potential of clays; η is viscosity; $n_{e}$ is effective porosity; and τ is tortuosity.

When the lower ends of the anode and cathode were both inserted 1 cm into the soil (mode *a*), and when the lower ends of the anode and cathode were in contact with the soil surface (mode *b*), the ions in the electrolyte found it difficult to be driven by the strongest electric field at the ends of the electrodes. Instead, they were mainly driven by the electric field created by the outer surfaces of the electrodes. Consequently, the number of migrating ions in the electrolyte is lower, resulting in a reduced cumulative volume of the anolyte.

The reason for the significantly lower reduction in anolyte volume during the E3 series of experiments is that flat EKG electrodes were used for the first 96 h of each experiment. The amount of conductive material in the flat EKG electrodes, as well as the cross-sectional area, was smaller compared to the tubular EKG electrodes. Consequently, the electric field strength generated was weaker than in the E1 and E2 series tests, which used tubular EKG electrodes under the same voltage, resulting in a lower reduction in the volume of the anolyte. Afterward, the electrodes in the E3 series of experiments were replaced with tubular EKGs, resulting in a significant increase in the current across the experiments. However, the reduction in the volume of the anode electrolyte did not increase significantly. This is because, during the initial 96 h of using the flat electrode, the current remained consistently below the threshold current of 15 mA, primarily leading to the occurrence of electrophoresis [30]. The prolonged electrophoresis in the early stage of E3 resulted in the pores between soil particles within a certain depth at the bottom of the anode chamber becoming densely filled, thereby affecting the transport of anolyte. As demonstrated by Sun et al. (2024) [20], the current in the electro-remediation process varied across different experiments under different voltage levels. In the experiment where the current remained consistently the highest, the corresponding decrease in the volume of the anolyte was certainly the greatest, and vice versa. However, in experiments where the current fluctuated, the relationship between the decreases in the anolyte volumes was relatively complex.

Due to factors such as experimental conditions, the heavy metal removal rate in this experimental scheme was not satisfactory. However, this did not affect the analysis of the effect of electrode position on the EKR effectiveness of contaminated soft clay with the electrolyte situated above the soil surface. In the process of EKR, the primary consideration is the removal of heavy metals. Electrode arrangement mode *c* can enhance the migration of the anolyte and reduce the drainage of the soil, making it an effective measure to improve the removal rate of heavy metals. After the remediation of heavy metals is completed, the bearing capacity of the soil should be increased. Changing the electrode arrangement to mode *d* is an effective measure to reduce the soil water content and improve soil strength.

## 4. Conclusions

The experimental results on the EKR effectiveness for Cu and Zn contamination in soft clay, both individually and in combination, are summarized below. These findings are based on a series of EKR experiments where the electrodes were positioned at various locations and the electrolyte was placed above the soil surface.

Electrode arrangement mode *c* (electrodes suspended in the electrolytes) can enhance the migration of the anolyte and reduce the drainage of the soil, making it an effective measure to improve the removal rate of heavy metals. After the remediation of heavy metals is completed, the bearing capacity of the soil should be increased. During this period, it is important to ensure that the current during electrochemical restoration remains above 15 mA to minimize the occurrence of electrophoresis. Prolonged electrophoresis in the early stages can lead to the pores between soil particles at a certain depth near the bottom of the anode chamber becoming densely packed, which can hinder the transport of the anolyte. For further development and utilization of remediated contaminated soft clay, changing the electrode arrangement to mode *d* is an effective measure to reduce soil water content and enhance soil strength. Furthermore, when the current was below 30 mA, the removal efficiency of heavy metal copper was slightly better than that of zinc. Under low current conditions, and with only a small amount of anolyte entering the contaminated soil, zinc tended to accumulate in the central and cathode regions of the soil, with particularly severe accumulation at the cathode.

## Figures and Tables

**Table 1:** Characteristics of clay powder used in this study.

Properties	Value
Particle size analysis	
Fine sand (%)	15
Silt (%)	49
Clay (%)	36
Water content (%)	5
pH	6.5
Liquid limit (%)	43
Plastic limit (%)	21
Permeability coefficient (cm/s)	4.0 × 10^−7^
Zn (mg/kg)	102 ± 2.90
Cu (mg/kg)	0 ± 0.34

**Table 2:** Experiment scenario for EKR tests.

Test Number	Heavy Metals (Concentration)	Applied Voltage (Duration)	Electrode Type	Total Processing Time (h)	Electrolyte (Concentration)
E1*a*	Cu (500 ± 10.26 mg/kg)	20 V (40 h)- 30 V (40 h)- 40 V (40 h)- 50 V (40 h)- 60 V (40 h)	Tubular EKG	200	CA (0.2 M)
E1*b*					
E1*c*					
E1*d*					
E2*a*	Zn (500 ± 15.51 mg/kg)				
E2*b*					
E2*c*					
E2*d*					
E3*a*	Cu and Zn (500 ± 19.07 mg/kg)	20 V (48 h)- 30 V (48 h)-20 V (40 h)- 30 V (40 h)- 40 V (21 h)- 50 V (16 h)- 60 V (33 h)	Flat and tubular EKG	246	
E3*b*					
E3*c*					
E3*d*					

*a* The lower ends of the electrodes in the electrolyte chamber were inserted into the soil to 1 cm. *b* The lower ends of the electrodes in the electrolyte chamber were in direct contact with the soil surface. *c* The length of the anode and cathode in the electrolytes was 8 cm. *d* The length of the anode in the anolyte was 8 cm, and the lower end of the cathode in the catholyte chamber was inserted into the soil to 1 cm.

**Table 3:** Comparison of the EKR test results under different experimental conditions.

Test Number	Change in Liquid Volume (mL)			Average Water Content (%)	Average Shear Strength (kPa)	Average Heavy-Metal Removal Rate (%)	
	Anolyte	Soil	Catholyte			Cu	Zn
E1*a*	−258.5 ± 0.9	430.1 ± 1.2	688.6 ± 1.9	36.7 ± 1.3	6.4 ± 0.5	23.0 ± 0.9	-
E1*b*	−134.4 ± 0.7	459.4 ± 1.3	593.8 ± 1.6	35.7 ± 1.3	6.8 ± 0.5	26.1 ± 1.3	-
E1*c*	−634.7 ± 1.7	298.8 ± 1.0	933.5 ± 2.9	39.4 ± 1.4	5.3 ± 0.4	45.2 ± 1.5	-
E1*d*	−237.5 ± 0.9	520.5 ± 1.4	758.0 ± 2.1	34.8 ± 1.3	9.1 ± 0.6	33.1 ± 1.2	-
E2*a*	−155.3 ± 0.7	449.9 ± 1.2	605.2 ± 1.6	38.0 ± 1.3	6.2 ± 0.5	-	−44.6 ± 1.4
E2*b*	−112.1 ± 0.6	470.7 ± 1.3	582.8 ± 1.5	37.0 ± 1.3	7.3 ± 0.5	-	−6.9 ± 0.3
E2*c*	−431.0 ± 1.2	310.5 ± 1.0	741.5 ± 2.0	40.5 ± 1.4	4.6 ± 0.4	-	12.3 ± 0.6
E2*d*	−206.5 ± 0.9	481.5 ± 1.3	688.0 ± 1.9	35.8 ± 1.3	8.6 ± 0.6	-	−4.2 ± 0.3
E3*a*	−59.5 ± 0.5	608.0 ± 1.6	667.5 ± 1.8	31.1 ± 1.2	13.7 ± 0.8	24.4 ± 0.8	−94.1 ± 2.6
E3*b*	−85.5 ± 0.5	561.0 ± 1.5	646.5 ± 1.7	30.3 ± 1.0	14.3 ± 0.8	36.7 ± 1.2	−17.4 ± 0.6
E3*c*	−63.4 ± 0.5	534.5 ± 1.4	597.9 ± 1.6	31.4 ± 1.2	13.9 ± 0.8	42.2 ± 1.3	−12.7 ± 0.5
E3*d*	−80.9 ± 0.5	521.3 ± 1.4	602.2 ± 1.6	31.7 ± 1.2	11.9 ± 0.7	31.1 ± 1.0	−143.3 ± 3.1

## Data Availability

The data presented in this study are available on request from the corresponding author.
